# Healthcare System Sustainability Challenges in Nepal and Opportunities Offered by Alternative Healthcare Delivery Arrangements

**DOI:** 10.31729/jnma.8584

**Published:** 2024-05-31

**Authors:** Pramila Rai, Denise A. O'Connor, Ilana N. Ackerman, Rachelle Buchbinder

**Affiliations:** 1 School of Public Health and Preventive Medicine, Monash University, Melbourne, Australia

**Keywords:** *delivery of health care*, *health care reform*, *Nepal*, *value-based health care*

## Abstract

The burden of chronic diseases in Nepal is increasing due to demographic and epidemiological transitions; alongside the persistent impact of communicable, maternal, newborn, and child health diseases, this critical situation acts as the precursor to rising healthcare costs. Nepal struggles to sustain its healthcare system amidst political instability, pandemics, natural disasters, and slow economic growth, particularly when healthcare funding is mainly dependent on out-of-pocket payments. Nepal requires lower-cost alternative healthcare delivery arrangements to provide high-value care while relieving economic sustainability pressures. Alternative healthcare delivery arrangements have a broad potential scope; they can involve strategic changes in how care is delivered and by whom, or they can also involve the application of information and communication technologies, e.g., telemedicine. This paper highlights the specific challenges to healthcare system sustainability in Nepal and the potential for high-value, lower-cost alternative healthcare delivery models to improve system performance in the longer term.

## INTRODUCTION

Healthcare services aim to improve people's health by providing affordable, accessible, efficient and effective healthcare.^1^ The financial sustainability of the healthcare system is of utmost importance to create a synchrony of healthcare components (including, for example, human resources, materials and infrastructure, and policy frameworks) to sustainably achieve this goal. Healthcare System sustainability is a concern for every country but poses a greater risk to low-income countries like Nepal.^2,3^ In this viewpoint, we elaborated on the factors that affect the sustainability of Nepal's healthcare system and propose considering high-value alternative healthcare delivery arrangements that could promote sustainability.

## WHAT ARE THE SPECIFIC CHALLENGES TO THE SUSTAINABILITY OF THE HEALTHCARE SYSTEM IN NEPAL?

**Macrostructure**

Nepal achieved political stability recently after transitioning into a federal system in 2017, following a decade-long People's War from 1996 to 2006 and an interim government.^4^ The delay in effective federalism impacted authority, responsibility, and accountability among governing and implementing agencies of the health sector. Poverty and disparities are also significant ongoing challenges with implications for the healthcare system. Additionally, unprecedented natural disasters such as the disastrous earthquake in April 2015 and the global coronavirus pandemic also worsened the country's progress in health and economic equality.

**Demand Domain**

The proportion of Nepalese over 65 years has increased from 4-2% in 2001 to 5.8% in 2019 and is expected to reach 7.1% of the total population by 2030.^5^ Chronic diseases, particularly cardiovascular disease andchronic obstructive pulmonary diseases, are the top two causes of death and disability in Nepal, surpassing the impact of infectious diseases.^6^ Increased longevity plus a higher prevalence of chronic diseases will likely lead to a greater number of years lived with disability in Nepal and a greater need for additional health and aged care services and increased healthcare expenditure. Nepal also faces disease outbreaks, floods, and landslides, which impact public health.^7^

**Supply Domain**

Nepal struggles to balance the supply and demand of healthcareproviders, resulting in a shortage of healthcare providers. The vacant positions of healthcare providers overall (26.6%) and of physicians/general practitioners (62.1%) in public health facilities in 2021 indicate a scarcity of healthcare workforce.^8^ On the other hand, Qualified nurses often face high unemployment rates, leading them to seek jobs abroad.^9^ This calls for extensive investment in planning, building and retaining the local healthcare workforce. The Nepal Health Facility Surveys also affirms the lack of trained staff for specific services, service-specific protocols, national standards or guidelines essential for high-quality clinical practice in most health facilities.^10^

**Economic Structure**

The healthcare budget steadily increases every year. The out-of-pocket payments constituted 54.2% of healthcare expenditure in 2019/20.^11^ As chronic diseases are associated with higher out-of-pocket payments, especially for prescription drugs,^12^ this would particularly impact the access of vulnerable population groups. Although basic healthcare services are subsidised by the Nepali government and provided at no or minimal cost to citizens in public health facilities, people opt for private health services as public health facilities commonly often lack medicines and other specialised services needed for comprehensive care.^13^ The national health insurance program implemented in 2016 is facing high drop-out rates of enrollees and providers, low renewals of premiums and low overall enrolment (only 21.4% in 2022) due to delayed or inadequate reimbursement to participating healthcare facilities, inadequate workforce and low-quality care for beneficiaries.^14^

## ARE ALTERNATIVE HEALTHCARE DELIVERY ARRANGEMENTS A POTENTIAL STRATEGY FOR IMPROVING THE SUSTAINABILITY OF NEPAL'S HEALTHCARE SYSTEM?

**Figure 1:**
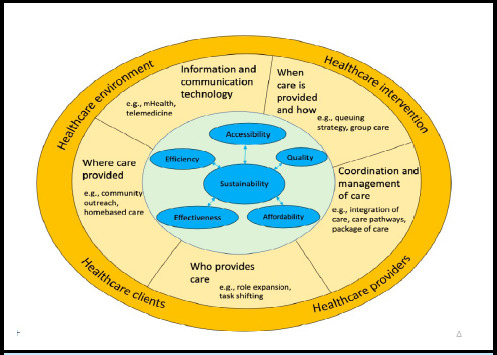
Alternative healthcare delivery of arrangements^15^ for the sustainability of the Nepali healthcare system.

The methodical integration of alternative healthcare delivery arrangements that provide more efficient and sustainable care may be one feasible strategy to improve healthcare performance without exhausting limited resources amidst those challenges (Figure 1).^15^ Alternative healthcare delivery arrangements refer to a strategic alteration in how healthcare services are organised and provided. These changes may involve healthcare providers, patients, settings or delivery formats. Cochrane Effective Practice and Organisation of Care (EPOC) categorises healthcare delivery arrangements into five subcategories: 'how and when care is delivered', 'where care is provided and changes to the healthcare environment', 'who provides care and how the healthcare workforce is managed','coordination of care and management of care and processes' and 'application of information communication technology'.

*The outer layer represents the healthcare ecosystem, where introducing alternative healthcare delivery arrangements (mid-layer) changes the healthcare system performance (inner layer).

A Cochrane overview published in 2016 brought attention to various delivery arrangements for different health conditions in other settings within these five subcategories.^3^ For instance, the available evidence suggests that alternative arrangements of task shifting and role expansion can lead to positive outcomes for health issues such as hypertension, maternal and child health and infectious diseases, abortion care, social support, and mental health. This approach could be valuable in Nepalese settings where access to care is limited by scarce specialised healthcare workforce and underutilisation of mid-level healthcare providers such as nurses. For example, task shifting of care of childbearing women and sick children at a community level to lay health workers and the integrated management of childhood illnesses have been applauded as effective strategies for reducing maternal, neonatal, and child mortality.^16^ The positive outcomes arising from these alternative models of care are encouraging and indicate the potential value of applying similar arrangements to deal with burgeoning chronic disease issues. One evidence gap is the need for cost-effectiveness data to support alternative delivery arrangements in countries such as Nepal. While many promising alternative delivery arrangements exist, such as care pathways and case management models, these have yet to be evaluated in low-income countries.^3^ Evaluating these arrangements and ensuring they are cost-effective and fit for purpose in the Nepali context is necessary.

## WAY FORWARD

In conclusion, the Nepali healthcare system faces multiple challenges threatening its future sustainability. Exploring and systematically adopting apt, effective, high-value, lower-cost alternative healthcare delivery arrangements may be a feasible strategy to promote healthcare system sustainability in Nepal.
